# Early tau detection in flortaucipir images: validation in autopsy-confirmed data and implications for disease progression

**DOI:** 10.1186/s13195-023-01160-6

**Published:** 2023-02-28

**Authors:** Vikas Kotari, Sudeepti Southekal, Michael Navitsky, Ian A. Kennedy, Ming Lu, Amanda Morris, Jennifer Ann Zimmer, Adam S. Fleisher, Mark A. Mintun, Michael D. Devous, Michael J. Pontecorvo

**Affiliations:** Eli Lilly and Company, Indianapolis, IN 46285 USA

## Abstract

**Background:**

There is an increasing interest in utilizing tau PET to identify patients early in Alzheimer’s disease (AD). In this work, a temporal lobe composite (*Eτ*) volume of interest (VOI) was evaluated in a longitudinal flortaucipir cohort and compared to a previously described global neocortical VOI. In a separate autopsy-confirmed study, the sensitivity of the *Eτ* VOI for identifying intermediate (B2) neurofibrillary tangle (NFT) pathology was evaluated.

**Methods:**

A total of 427 subjects received flortaucipir, florbetapir, MRI, and cognitive evaluation at baseline and 18 months. In a separate autopsy study, 67 subjects received ante-mortem flortaucipir scans, and neuropathological findings were recorded according to NIA-AA recommendations by two experts. Two VOIs: *Eτ* comprising FreeSurfer volumes (bilateral entorhinal cortex, fusiform, parahippocampal, and inferior temporal gyri) transformed to MNI space and a previously published global AD signature-weighted neocortical VOI (AD_signature_) (Devous et al., J Nucl Med 59:937–43, 2018), were used to calculate SUVr relative to a white matter reference region (PERSI) (Southekal et al., J Nucl Med Off Publ Soc Nucl Med 59:944–51, 2018). SUVr cutoffs for positivity were determined based on a cohort of young, cognitively normal subjects. Subjects were grouped based on positivity on both VOIs (*Eτ+*/AD_signature_+; *Eτ+*/AD_signature_–; *Eτ*−/AD_signature_−). Groupwise comparisons were performed for baseline SUVr, 18-month changes in SUVr, neurodegeneration, and cognition. For the autopsy study, the sensitivity of *Eτ* in identifying intermediate Braak pathology (B2) subjects was compared to that of AD signature-weighted neocortical VOI. The average surface maps of subjects in the *Eτ+*/AD_signature_− group and B2 NFT scores were created for visual evaluation of uptake.

**Results:**

Sixty-four out of 390 analyzable subjects were identified as *Eτ+*/AD_signature_–: 84% were Aβ+, 100% were diagnosed as MCI or AD, and 59% were *APOE ε4* carriers. Consistent with the hypothesis that *Eτ+*/AD_signature_– status reflects an early stage of AD, *Eτ+*/AD_signature_– subjects deteriorated significantly faster than *Eτ–*/AD_signature_*–* subjects, but significantly slower than *Eτ+*/AD_signature_+ subjects, on most measures (i.e., change in AD_signature_ SUVr, *Eτ* ROI cortical thickness, and MMSE). The AD_signature_ VOI was selective for subjects who came to autopsy with a B3 NFT score. In the autopsy study, 12/15 B2 subjects (including 10/11 Braak IV) were *Eτ+*/AD_signature_–. Surface maps showed that flortaucipir uptake was largely captured by the *Eτ* VOI regions in B2 subjects.

**Conclusion:**

The *Eτ* VOI identified subjects with elevated temporal but not global tau (*Eτ+*/AD_signature_–) that were primarily Aβ+, *APOE ε4* carriers, and diagnosed as MCI or AD. *Eτ+*/AD_signature_– subjects had greater accumulation of tau, greater atrophy, and higher decline on MMSE in 18 months compared to *Eτ*−/AD_signature_− subjects. Finally, the *Eτ* VOI identified the majority of the intermediate NFT score subjects in an autopsy-confirmed study. As far as we know, this is the first study that presents a visualization of ante-mortem FTP retention patterns that at a group level agree with the neurofibrillary tangle staging scheme proposed by Braak. These findings suggest that the *Eτ* VOI may be sensitive for detecting impaired subjects early in the course of Alzheimer’s disease.

**Supplementary Information:**

The online version contains supplementary material available at 10.1186/s13195-023-01160-6.

## Key points

- Question: Can a flortaucipir VOI made up of temporal lobe structures (*Eτ* VOI) identify patients early in the course of AD?
- Pertinent findings: The *Eτ* VOI identified subjects with elevated temporal, but not global flortaucipir PET signal who were primarily Aβ+, *APOE ε4* carriers, and diagnosed as MCI or AD. The identified patients demonstrated modest tau accumulation, neurodegeneration, and cognitive decline within 18 months. Finally, the *Eτ* VOI identified the majority of the amyloid-positive, Braak IV (intermediate ADNC score) subjects in an autopsy-confirmed study.
- Implications for patient care: In research settings, the early tau VOI might aid in the identification and stratification of patients early in the course of AD who may derive maximal benefit from therapeutic intervention.

## Introduction

The defining neuropathological hallmarks of Alzheimer’s disease (AD) are an extracellular accumulation of aggregated amyloid-*β* fragments, typically associated with degenerating neurites (amyloid-β neuritic plaques), and intracellular aggregates of abnormally phosphorylated microtubule-associated tau protein (neurofibrillary tangles [NFTs]) [1, 2]. NFTs may be found within the transentorhinal cortex and nearby mesial temporal lobe structures of both healthy older adults and subjects with Alzheimer’s disease dementia [3]. However, the widespread presence of NFTs in the neocortex is typically associated with cognitive impairment [4]. It is hypothesized that in individuals on the Alzheimer’s disease pathway, amyloid plaques (Aβ) trigger the spread of neurofibrillary tangles beyond the transentorhinal cortex [5, 6].

With the advancement of positron emission tomography (PET) imaging biomarkers for amyloid (the approved agents florbetapir F 18, florbetaben F 18, and flutemetamol F 18) and tau (the approved agent flortaucipir F 18 as well as experimental agents such as ^18^F-MK-6240 and ^18^F-PI-2620), the density and distribution of misfolded amyloid beta and hyperphosphorylated tau aggregates can be estimated in vivo [7–12]. Fleisher et al. [13] have recently shown in an autopsy-confirmed dataset that a specific pattern of flortaucipir neocortical retention was associated with both Braak V/VI stage or B3 NFT score and Aβ accumulation, which is consistent with high AD neuropathological change according to the NIA-AA criteria [4, 14].

Recent studies have also shown a correlation between flortaucipir estimation of tau burden and increasing progression of cognitive impairment even when the analysis is limited to amyloid-positive subjects [15–17]. These findings suggest that moderate to advanced tau burden as estimated by flortaucipir might help confirm AD diagnosis and identify patients most likely to deteriorate within a subsequent 18-month time period.

In addition to confirming the presence of AD pathology, there has been increasing interest in utilizing tau PET imaging to identify patients at the earliest stages of AD [18–20]. Braak et al. have suggested that tau starts in the transentorhinal cortex, spreads to the entorhinal cortex, and then extends into the fusiform and temporal gyri before spreading to the parietal, occipital, and frontal cortices [21, 22]. Multiple neuropathological studies report on the presence of NFTs in the medial temporal lobes of cognitively normal subjects [3, 23, 24]. Increased tracer retention has been shown in the temporal regions of cognitively normal, amyloid-positive, and amyloid-negative subjects using tau PET imaging [17, 19, 25]. Composite temporal volumes-of-interest (VOIs) to quantify early AD subjects from tau PET images have been previously proposed [26–29]. In this work, we hypothesized that a similar temporal composite VOI for flortaucipir imaging comprising medial/ inferior temporal lobe structures might identify subjects that may be in the earliest stages of AD tauopathy before widespread tau accumulation. We applied this VOI in two longitudinal clinical trials, as well as a separate autopsy validation study to identify subjects with tau limited to the temporal regions. For the two longitudinal studies, we also examined the changes in tau accumulation, neurodegeneration, and cognition over 18 months associated with baseline temporal VOI findings. The main goal of our work was the identification and disease progression of early tau subjects that might not have global tau uptake. In the autopsy-confirmed dataset, we evaluated the sensitivity of the VOI for identifying intermediate Braak stage pathology.

## Methods

### Subjects

#### Longitudinal study 1 and study 2 (18-F-AV1451-A05 exploratory and Expedition 3)

We retrospectively analyzed 427 subjects from our exploratory longitudinal study of flortaucipir (A05-exploratory, NCT02016560) and a previously completed therapeutic trial where flortaucipir was used as a biomarker (Expedition 3/NCT01900665). In both trials, subjects received flortaucipir, florbetapir, anatomical magnetic resonance imaging (T_1_-weighted MRI), and a battery of cognitive assessments both at baseline and approximately at 18 months. Some of the Expedition 3 trial participants received solanezumab as treatment; all the A05 subjects were untreated. None of the participants received solanezumab treatment prior to baseline flortaucipir or florbetapir scans. The demographics of all these subjects are shown in Table 1.

**Table 1:** Demographics of subjects that were retrospectively analyzed in this work. Age is represented as mean ± standard deviation. MMSE is also represented as mean ± standard deviation. All the 109 subjects who received solanezumab were amyloid-positive and mild to moderate AD cases. *YCN* young cognitively normal, *CN* cognitively normal, *MCI* mild cognitive impairment, *AD* Alzheimer’s disease, *N* number of subjects, *MMSE* Mini-Mental State Examination, *F* female, *M* male. *CD* college degree or higher, *HS* high school or lower, *C* Caucasian, *NC* Not Caucasian (Asian, Black or African American, Other)

	**YCN/Aβ-**	**CN/Aβ-**	**CN/Aβ+**	**MCI/Aβ-**	**MCI/Aβ+**	**AD/Aβ-**	**AD/Aβ+**
**N**	16	53	5	50	47	16	240
**Age**	28.9 ± 4.9	67.6 ± 10.2	77.8 ± 7.0	69.1 ± 9.3	72.7 ± 9.1	72.2 ± 6.9	73.7 ± 8.3
**MMSE**	29.6 ± 0.5	29.5 ± 0.5	29.6 ± 0.5	28.2 ± 1.7	27.4 ± 1.8	22.9 ± 3.2	22.5 ± 2.9
**Sex**	7F/9M	24F/29M	2F/3M	27F/23M	21F/26M	7F/9M	135F/104M
**Education**	15CD/1HS	45CD/8HS	4CD/1HS	37CD/13HS	38CD/9HS	14CD/2HS	141CD/97HS
**Race**	11C/5NC	42C/11NC	5C/0NC	43C/7NC	45C/2NC	14C/2NC	192C/22NC

#### Study 3 (18-F-AV1451-A16)

Sixty-seven subjects with a terminal illness, a projected life expectancy of less than 6 months, and older than 50 years underwent ante-mortem flortaucipir PET imaging [13]. These subjects were then followed with brain autopsy (18-F-AV1451-A16, NCT02516046). Two pathologists blinded to the clinical and imaging results recorded neuropathological findings according to the NIA-AA guidelines [4, 14]. Braak pathological staging of NFTs was performed by AT8 monoclonal antibody, and Aβ plaques were detected using 6E10 Aβ_1–42_ monoclonal antibody using immunohistochemical staining methods [13]. The demographics of these subjects are shown in Table 2.

**Table 2:** Demographics of the autopsy-confirmed subjects, where *N* is the number of subjects, age is represented as mean ± SD, and gender is shown as female/male. *CD* college degree or higher, *HS* high school or lower, *C* Caucasian, *NC* Not Caucasian (Asian, Black or African American, Other)

	**B1**		**B2**		**B3**	
	**Braak I**	**Braak II**	**Braak III**	**Braak IV**	**Braak V**	**Braak VI**
**N**	2	5	4	11	14	24
**Age**	67 ± 1.7	76.8 ± 6.8	85.8 ± 10.4	86.8 ± 6.3	85.6 ± 8.5	81.7 ± 9.4
**Sex**	1F/ 1M	3F/ 2M	3F/ 1M	6F/ 5M	5F/ 9M	15F/ 9M
**Education**	1CD/1HS	2CD/3HS	3CD/1HS	4CD/7HS	11CD/3HS	14CD/10HS
**Race**	2C/0NC	4C/1NC	4C/0NC	11C/0NC	14C/0NC	24C/0NC

### Image acquisition

#### Study 1 (NCT02016560/18-F-AV1451-A05 exploratory)

Flortaucipir images were acquired for 20 min (4 × 5 minute frames) from 80 to 100 min post-injection of approximately 370 MBq (10mCi) of flortaucipir F 18. On a separate day, 10-min florbetapir images were acquired between 50 and 60 min post-injection of approximately 370 MBq (10mCi) of florbetapir F 18. PET data were reconstructed using scanner-specific iterative reconstruction algorithms (FORE, OSEM, or RAMLA) with 3–6 iterations, 16–33 subsets, and post-smoothing of 3–5 mm (or “normal” or “sharp” settings).

Each subject also underwent a 3D T_1_-weighted MRI scan on either a 1.5-T or 3-T scanner. The MRI protocol included sagittal 3D MPRAGE (Siemens), sagittal 3D T1-TFE (Philips), or sagittal 3D IR-FSPGR (GE) sequences with 1.2-mm-thick slices (no gap) and 0.9375–1.25 × 0.9375–1.25 mm^2^ in-plane resolution.

#### Study 2 (NCT01900665/Expedition 3)

Flortaucipir images were acquired for 30 min (6 × 5 minute frames) from 75 to 105 min post-injection of 240 MBq (6.5 mCi) of flortaucipir F 18. The reconstruction algorithms were similar to study 1. All subjects also underwent florbetapir PET scans 50–70 min after injection of 370 MBq florbetapir F 18 and were required to be amyloid-positive by visual interpretation of PET at screening. The MRI acquisition parameters were the same as in study 1.

#### Study 3 (NCT02516046/18-F-AV1451-A16)

For this autopsy study in end-of-life patients, flortaucipir acquisition parameters were the same as in study 1. No florbetapir or MRI data were acquired. Amyloid positivity was determined by a CERAD score of moderate to frequent at autopsy.

### Image processing and interpretation

#### Florbetapir (A05E)

For each subject, the florbetapir PET frames were motion-corrected (MCFLIRT [30]) and averaged to create a single static image. Florbetapir static images were spatially aligned to the Montreal Neurological Institute (MNI) template and normalized using the whole brain cerebellum to create an SUVR image [31]. The average SUVR from six predefined ROIs was calculated. Visual assessment of the florbetapir images supplemented by SUVR was used to determine Aβ positivity [32].

#### Flortaucipir (studies 1 and 2)

Dynamic flortaucipir PET frames were corrected for inter-frame motion (McFLIRT [30]) and acquisition start time deviations prior to averaging to create a static image. The mean PET static baseline flortaucipir image for a given subject was rigidly aligned to the subject’s baseline MRI. Follow-up flortaucipir images were aligned to the baseline PET. A nonlinear transformation of the subject’s baseline MRI to the MNI template was conducted. This non-linear transformation was applied to the native space-aligned flortaucipir PET images from all time points. A subject-specific, data-driven, white matter reference region (PERSI) [33] was used for count normalization. In addition to PERSI, we evaluated the performance of previously published cerebellum crustaneous as a reference region. We excluded 37 subjects from further analyses due to severe motion that affected quantitation and image artifacts or difficulties identifying a separable white matter peak for the reference region that led to failures in quantification.

#### Flortaucipir (study 3)

Dynamic flortaucipir images from study 3 were corrected for motion and acquisition start time deviations prior to creating static images, similar to studies 1 and 2. Since subjects in this study did not receive an MRI, the PET image was directly aligned to a flortaucipir PET template. This flortaucipir PET template was created by averaging previously aligned MNI space flortaucipir PET images from study 1. Seven subjects from this study were excluded from further analyses due to image artifacts or severe motion.

#### Visual interpretation

Flortaucipir images from all three studies were visually read by imaging physicians as either not consistent with AD (negative AD tau pattern/*τ*AD−) or consistent with AD (moderate/*τ*AD+, or advanced/*τ*AD++ tau pattern), based on neocortical tracer pattern [34].

### Analysis

#### Early tau VOI

**Fig. 1:**
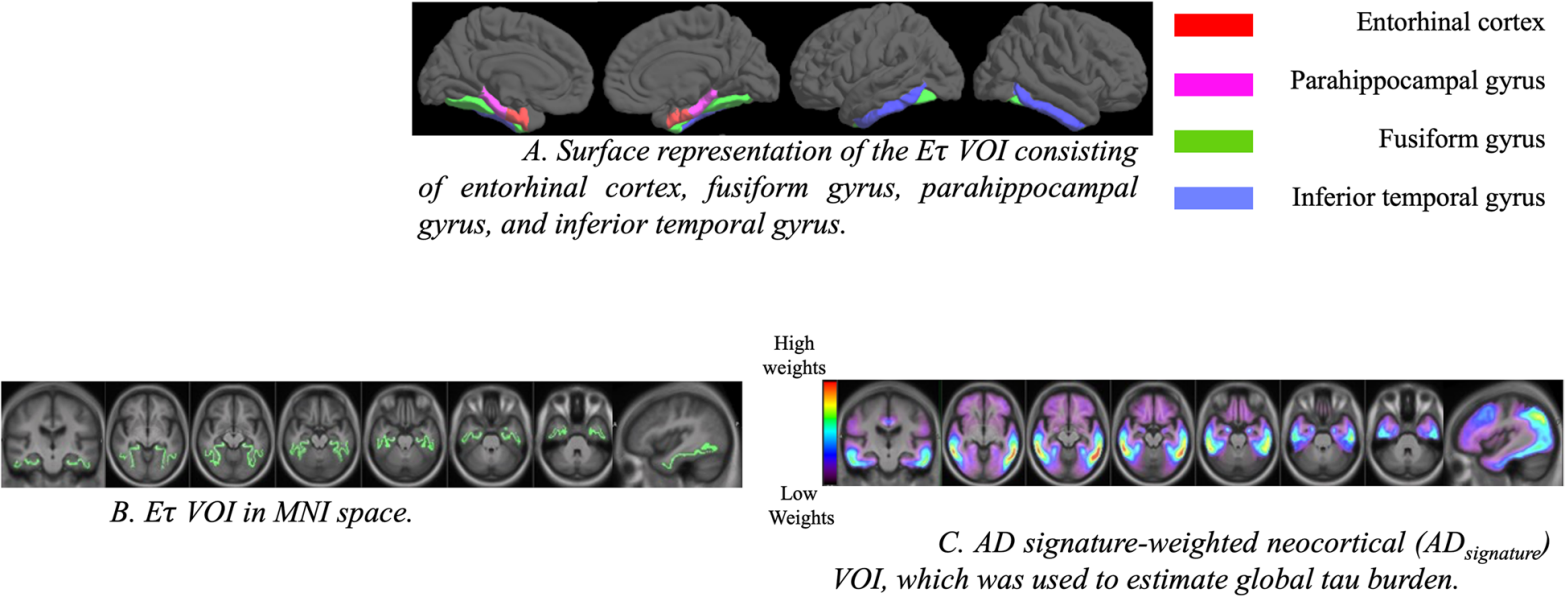
**A** Surface representation of the *Eτ* VOI consisting of the entorhinal cortex, fusiform gyrus, parahippocampal gyrus, and inferior temporal gyrus. **B** *Eτ* VOI in MNI space. **C** AD signature-weighted neocortical (AD_signature_) VOI, which was used to estimate global tau burden

Based on preliminary analyses performed to separate Aβ+ controls from Aβ− controls and evidence in the literature of early involvement of temporal lobe structures in tau accumulation [19, 25, 26, 28, 35], we selected entorhinal cortex, parahippocampal, fusiform, and inferior temporal gyri to create an “early tau” VOI (*Eτ* VOI) to capture what we hypothesize would be the region of early flortaucipir uptake. We transformed the FreeSurfer [36] FsAverage template to the MNI space and extracted the volumes based on the Desikan-Killiany atlas [37] to create the VOI. Figure 1A is a surface representation of the *Eτ* VOI. Figure 1B is the volumetric representation of the *Eτ* VOI, which was applied to all the subjects.

#### AD signature-weighted neocortical VOI for the estimation of global tau burden

In order to estimate the global tau burden, we used a data-driven volume of interest that was designed to maximize separation between diagnostic groups (Aβ+ AD, Aβ+ mild cognitive impairment [MCI] vs dementia, MCI, and cognitive normal [CN] Aβ− subjects) using a multiblock barycentric discriminant analysis (MUBADA) [38]. As shown in Fig. 1C, the AD signature-weighted neocortical VOI (AD_signature_) [39] has higher weights in the posterolateral temporal and parietal regions.

#### SUVr analysis and subject grouping

**Fig. 2:**
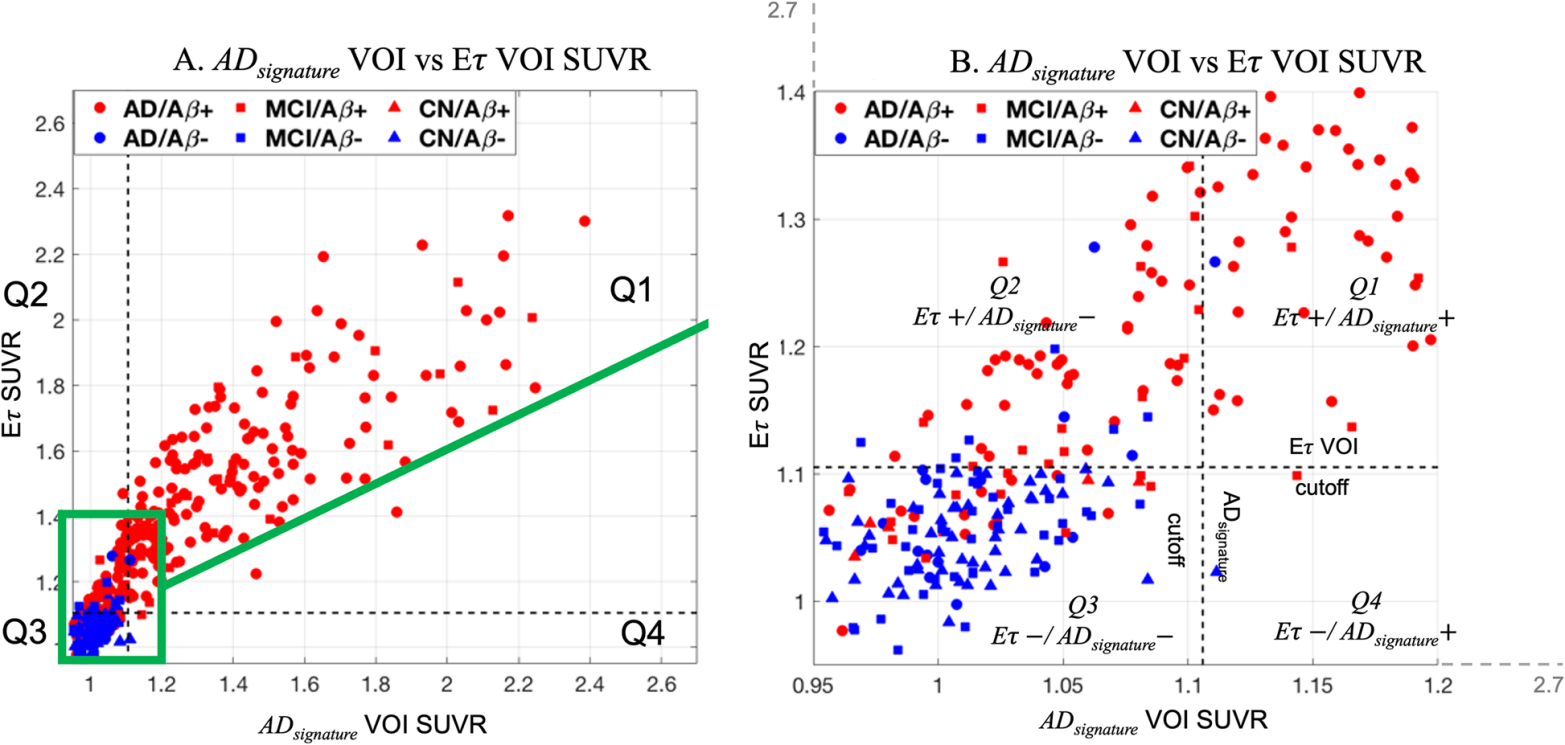
**A** Scatter plot showing AD_signature_ SUVR vs *Eτ* VOI SUVR with subjects color-coded based on amyloid status (Aβ+ is red and Aβ− is blue). **B** A zoomed sub-section of **A** highlighting the subjects in the *Eτ+*/AD_signature_– quadrant

The *Eτ* VOI and AD_signature_ VOI SUVRs were calculated relative to a white matter reference region (PERSI) [33]. Sixteen cognitively normal, young (age = 28.9 ± 4.9) subjects from study 1 were used to determine SUVR thresholds for positivity for each volume of interest. Thresholds for positivity were defined as the mean + 2.5 times standard deviation of SUVRs from these sixteen young controls. Based on SUVR thresholds within the *Eτ* VOI (1.1052) and AD_signature_ VOI (1.1059), subjects were divided into quadrants of elevated or low tau signal (Fig. 2). Subjects elevated in both *Eτ* and AD_signature_ VOIs were represented as *Eτ+*/AD_signature_+, subjects positive for *Eτ* alone were *Eτ+*/AD_signature_–, subjects with low *Eτ* and AD_signature_ were *Eτ*−/AD_signature_−, and subjects with low *Eτ* and high AD_signature_ were *Eτ*−/AD_signature_+. Since only two subjects were *Eτ*−/AD_signature_+, we did not include them in any analysis. Among these groups, we were particularly interested in *Eτ+*/AD_signature_– subjects as this sub-group represented subjects with tau uptake primarily restricted to the regional *Eτ* VOI, but below the threshold for the global AD signature-weighted neocortical VOI.

#### Cortical thickness

Each subject’s baseline and 18-month T_1_-weighted MRI was processed in FreeSurfer version 5.3 [36] to generate subject-specific regional cortical thickness measures. Subjects with image or motion artifacts in MRI were excluded from cortical thickness assessments. Since we do not expect tau uptake in *Eτ* VOI to impact neurodegeneration outside of the early tau region, we calculated vertex-weighted average thickness from the *Eτ* VOI regions (entorhinal cortex, fusiform, parahippocampal, and inferior temporal gyri) using the Desikan-Killiany atlas for each subject. We calculated the difference in thickness between the two time points within the *Eτ* VOI regions to capture cortical thinning.

#### Statistical analysis

To understand whether subjects with elevated *Eτ* VOI (temporal) signal and low AD signature-weighted neocortical VOI (global) signal, i.e., *Eτ+*/AD_signature_– subjects, were likely to progress, we performed quadrant-wise statistical analyses. Subjects that were *Eτ+*/AD_signature_+, *Eτ+*/AD_signature_–, and *Eτ*−/AD_signature_− were compared for tau accumulation in *Eτ* and AD_signature_ VOI, cortical atrophy within *Eτ* regions, and cognition. To eliminate any treatment-related confounds, we excluded 109 subjects from Expedition 3 trial that received solanezumab from all longitudinal statistical analyses.

We calculated the baseline and 18-month change in *Eτ* VOI SUVR and AD signature-weighted neocortical SUVR for each subject to assess tau accumulation. The Kruskal-Wallis test was performed to assess the group differences across quadrants. Post hoc tests were performed between each of the three quadrants. Similar to the change in tau accumulation, we performed group comparisons and post hoc analyses for baseline cortical thickness and 18-month change in thickness.

For evaluating cognitive decline, we compared the 18-month change in Mini-Mental State Examination (MMSE) across quadrants. We also compared the 18-month change in Pfeffer Functional Activities Questionnaire (FAQ) and Alzheimer’s Disease Assessment Scale 11-item Cognitive Subscale (ADAS-Cog11).

#### Validation in autopsy data

The *Eτ* and AD signature-weighted neocortical VOI SUVRs were also derived for subjects in study 3. Scatter plots of the autopsy-confirmed Braak stage against the SUVR were used to assess the number of subjects in each Braak stage above the cutoffs for *Eτ* and AD_signature_ VOIs.

#### Neocortical flortaucipir uptake

In order to explore whether the *Eτ+*/AD_signature_– subjects have elevated tau uptake in other regions besides the *Eτ* VOI, we created quadrant-wise average surface maps for each of the three quadrants. Each quadrant’s average image was projected onto the FsAverage surface.

Voxel-wise *z*-scores were determined for each subject relative to SUVR values from the sixteen young controls from study 1. For each voxel, group-wise average *z*-scores were measured by grouping subjects within the previously identified quadrants *Eτ+*/AD_signature_+, *Eτ+*/AD_signature_–, and *Eτ*−/AD_signature_−. If the average *z*-score of a voxel was at least 2, it was considered as being tau positive. In order to observe if tau positivity in a voxel was present in multiple subjects, we also calculated the percentage of tau-positive (*z*-score > 2) subjects within each voxel.

The same approach was applied to study 3 (autopsy-confirmed dataset) to create average surface maps based on NFT scores.

## Results 

### Subject distribution

Of all the baseline subjects in this study, 171 subjects showed elevated signal in both *Eτ* VOI and AD_signature_ VOI (*Eτ+*/AD_signature_+), 64 subjects were identified as having elevated *Eτ* VOI signal in the absence of AD_signature_ VOI signal (*Eτ+*/AD_signature_–), 153 subjects had low signal on both *Eτ* and AD_signature_ VOI (*Eτ*−/AD_signature_−), and 2 subjects were high on AD_signature_ and low on *Eτ* VOI (*Eτ*−/AD_signature_+) (Fig. 2A, B). *Eτ+*/AD_signature_+ subjects were 99% Aβ+, 100% diagnosed as either AD (88%) or MCI (12%), and 74% were *APOE ε4* carriers. Eτ+/AD_signature_– subjects were 84% Aβ+, 100% were either AD (67%) or MCI (33%), and 59% were *APOE ε4* carriers. In contrast, *Eτ*−/AD_signature_− subjects were only 19% Aβ+, 18% AD (40% MCI), and 30% *APOE ε4* carriers. Since all subjects in study 2 were Aβ+ and mild to moderate AD, we also calculated percentages in study 1. We observed that 98% of subjects in *Eτ+*/AD_signature_+, 68% of subjects in *Eτ+*/AD_signature_−, and 13% of subjects in *Eτ*−/AD_signature_− were Aβ+ in study 1. In terms of *APOE ε4* carrier status, 73% of *Eτ+*/AD_signature_+ subjects, 50% of *Eτ+*/AD_signature_− subjects, and 29% of *Eτ*−/AD_signature_− subjects were *APOE ε4* carriers. These results are shown in Additional file 5: Table S1.

### SUVR, thickness, and cognition/function

**Fig. 3:**
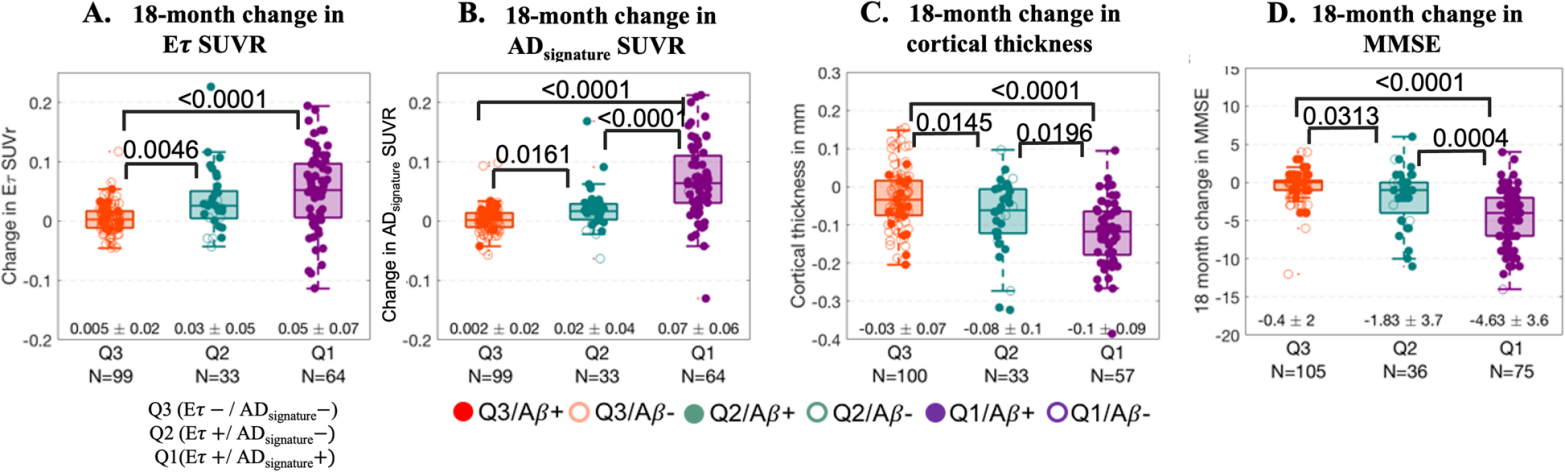
**A** Boxplots showing the 18-month change in *Eτ* VOI SUVR across the quadrants. The 18-month change in *Eτ* VOI SUVR was significantly different between subjects in Q2 (or Q1) and Q3. **B** Boxplots showing the 18-month change in AD_signature_ VOI SUVR across the quadrants. The 18-month change in AD_signature_ VOI SUVR was significantly different across the quadrants (Q1 > Q2 > Q3). **C** Boxplots showing the 18-month change in cortical thickness across the quadrants. The 18-month change in cortical thickness was significantly different across the quadrants (Q1 < Q2 < Q3). **D** Boxplot showing the 18-month change in MMSE across the quadrants. The 18-month change in MMSE was significantly different across quadrants (Q1 < Q2 < Q3). A total of 109 subjects from the treatment arm of the solanezumab trial were excluded from these analyses. *Note*: the *N*’s are different in the above boxplots for flortaucipir change, thickness, and MMSE due to the availability of the data

There was a significant difference in baseline flortaucipir SUVR between *Eτ+*/AD_signature_+, *Eτ+*/AD_signature_−, and *Eτ*−/AD_signature_− for both *E*𝜏 and AD_signature_ VOIs (Fig. S1). Elevation in flortaucipir retention in the *E*𝜏 VOI had consequences for future changes in SUVR and cortical thickness in the early tau region, as well as for cognitive deterioration. Compared to *Eτ*−/AD_signature_− subjects, *Eτ+*/AD_signature_– and *Eτ+*/AD_signature_+ had significantly greater SUVR increases over 18 months in both E𝜏 (Fig. 3A) and AD_signature_ (Fig. 3B) regions. *Eτ+*/AD_signature_+ subjects had greater SUVR increases than *Eτ+*/AD_signature_– subjects in the AD_signature_ VOI (Fig. 3B). However, for the *E*𝜏 VOI, there was no difference in SUVR increase between the *Eτ+*/AD_signature_– and *Eτ+*/AD_signature_+ subjects (Fig. 3A).

Similar differences were seen for the changes in cortical thickness within the *Eτ* VOI (Fig. 3C), among the three quadrants. Likewise, significant differences were observed between all quadrants for 18-month change in MMSE (Fig. 3D). There was also a significant difference between *Eτ*−/AD_signature_− and *Eτ+*/AD_signature_– or *Eτ+*/AD_signature_+ subjects for 18-month change in ADAS-Cog11 and FAQ. However, differences in the change from baseline ADAS-Cog11 or FAQ between *Eτ+*/AD_signature_– and *Eτ+*/AD_signature_+ subjects did not reach statistical significance.

We observed that using the cerebellum crustaneous reference region resulted in fewer subjects being identified as *Eτ+*/AD_signature_–. While the subjects identified using cerebellum crustaneous had on average greater tau accumulation, larger cortical thinning, and higher cognitive decline in 18 months, they were not significantly different from *Eτ*−/AD_signature_− group (Fig. S2A-D).

### Validation in autopsy-confirmed dataset

**Fig. 4:**
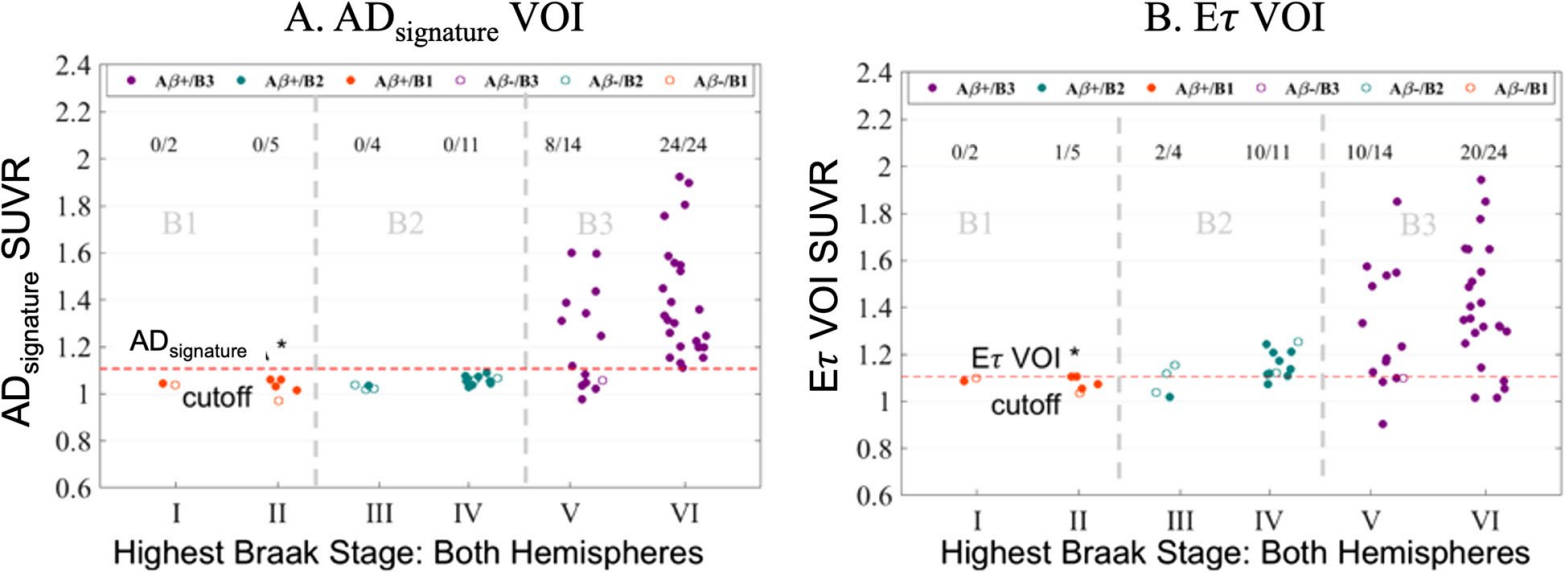
**A** Scatter plot of Braak stage vs AD_signature_ VOI SUVR. The red dotted line represents the AD_signature_ positivity cutoff of 1.1059. The numbers at the top represent the number of subjects for each Braak stage. For example, for Braak VI, 24/24 indicates that of the 24 Braak VI subjects in this analysis, 24 had elevated AD_signature_ signals. **B** Scatter plot of Braak stage vs *Eτ* VOI SUVR. The red dotted line represents the *Eτ* VOI SUVR positivity cutoff of 1.1052. Amyloid positivity is defined by a CERAD score of moderate to frequent at autopsy

In the autopsy-confirmed data, 32 of the 38 B3 NFT score subjects had elevated AD_signature_ VOI signal (Fig. 4A), and 30/38 B3 NFT score subjects had elevated *Eτ* VOI signal (Fig. 4B) as seen in the scatter plots (Fig. 4A, B). In contrast, none of the 15 B2 NFT score subjects had elevated AD_signature_ VOI signal, but 12/15 (including 10/11 Braak IV) had elevated *Eτ* VOI signal (Fig. 4B). None of the B1 NFT score subjects had elevated AD_signature_ VOI signal, one B1 (Braak II) NFT score subject had elevated *Eτ* VOI signal.

**Fig. 5:**
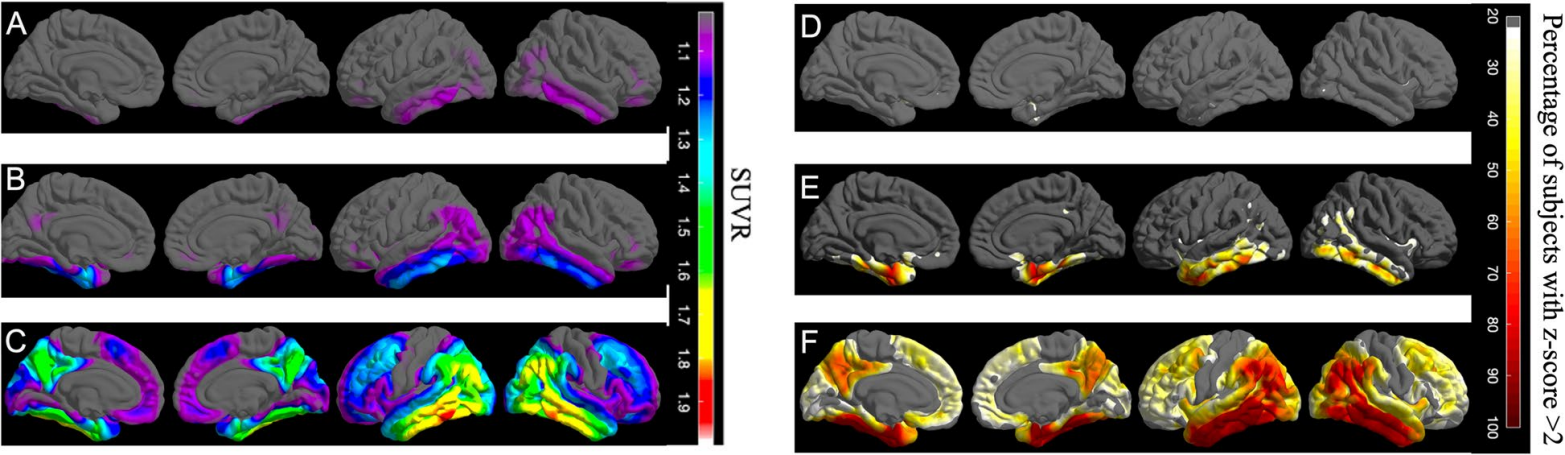
**A** The average SUVR surface map of Q3 (*Eτ*−/AD_signature_−) subjects, showing a lack of elevated flortaucipir signal in the entire cortex. **B** The average SUVR surface map of Q2 (*Eτ+*/AD_signature_–) subjects. There is an elevated flortaucipir signal primarily in the regions of *Eτ* VOI region with the exception of the left middle temporal gyrus. **C** The average SUVR surface map of Q1 (*Eτ+*/AD_signature_+) subjects. Widespread elevation of flortaucipir signal across the entire cortex with the exception of postcentral, precentral, and paracentral gyri and anterior cingulate. **D**–**F** Surface maps showing the percentages of subjects that are tau positive for all FreeSurfer regions in Q3 (*Eτ*−/AD_signature_−), Q2 (*Eτ+*/AD_signature_–), and Q1 (*Eτ+*/AD_signature_+), respectively

With cerebellum crustaneous reference region, 31/38 B3 NFT score subjects had elevated AD_signature_ and *Eτ* VOI signal. Three of the 15 B2 NFT score subjects had elevated AD_signature_ signal but 7/15 B2 NFT score subjects had elevated *Eτ* VOI signal. None of the B1 NFT score subjects had elevated AD_signature_ VOI signal, while one B1 (Braak II) NFT score subject had an elevated *Eτ* VOI signal (Fig. S3A, B).

### Neocortical tau burden

There were no vertices of elevated flortaucipir uptake in *Eτ*−/AD_signature_− subjects (Fig. 5A). For *Eτ+*/AD_signature_– subjects, as hypothesized, elevated uptake was primarily restricted to the *Eτ* VOI, with the exception of left middle temporal gyrus (Fig. 5B). *Eτ+*/AD_signature_+ subjects had elevated uptake across the entire cortex sparing the precentral, postcentral, and paracentral gyri and the anterior cingulate (Fig. 5C).

Figure 5D–F shows the percentage of subjects with regional tau positivity for *Eτ*−/AD_signature_−, *Eτ+*/AD_signature_–, and *Eτ+*/AD_signature_+, respectively. None of the *Eτ*−/AD_signature_− subjects were tau-positive in any of the regions sampled at the selected threshold. Within the *Eτ* VOI, 70% of *Eτ+*/AD_signature_– subjects were tau-positive, with 90.7% positive in the left entorhinal cortex, 87.5% in the right entorhinal cortex, 92.2% in the left inferior temporal gyrus, and 67.2% in the right inferior temporal gyrus. Within the neo-cortex, outside the *Eτ* VOI, 46.8% of subjects in *Eτ+*/AD_signature_– were positive in the left middle temporal gyrus, 35.9% in the right middle temporal gyrus, and only 28.1% in the right inferior parietal gyrus. For *Eτ+*/AD_signature_+ subjects, the percentage of tau positivity was highest (~ 90%) within the medial/lateral temporal including the *Eτ* VOI and inferior parietal structures, followed by superior temporal, superior parietal, and supramarginal regions (~ 60%) and frontal regions (~ 50%).

**Fig. 6:**
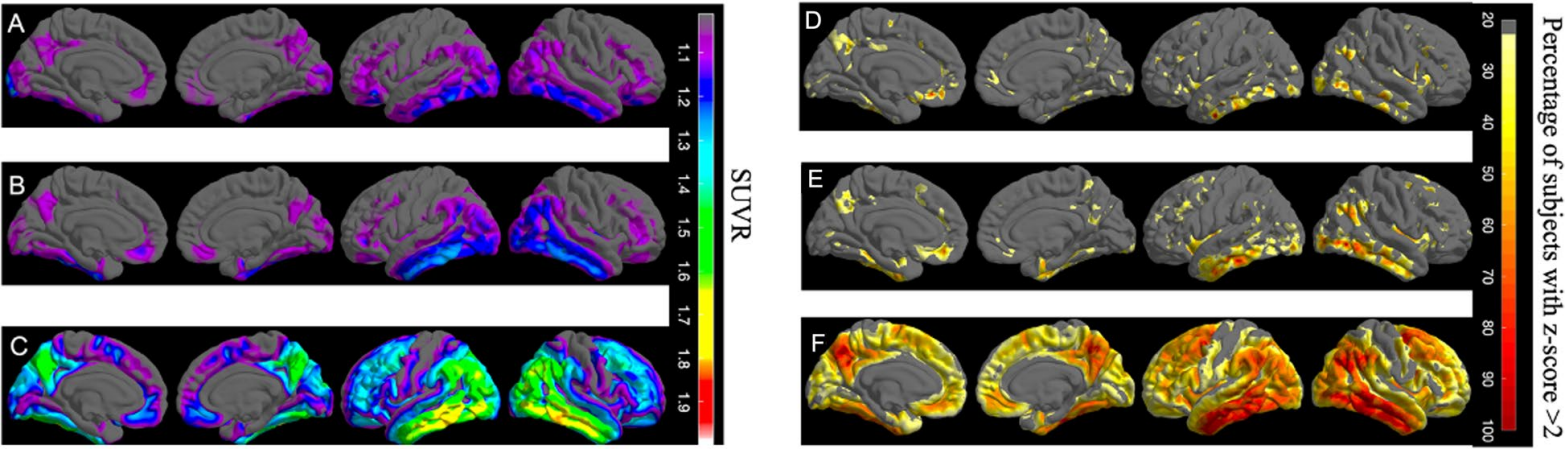
**A** The average SUVR surface map of B1 subjects, showing a lack of elevated flortaucipir signal in the entire cortex. **B** The average SUVR surface map of B2 subjects. There is an elevated flortaucipir signal primarily in the regions of *Eτ* VOI region with the exception of the left middle temporal gyrus. **C** The average SUVR surface map of B3 subjects. Widespread elevation in flortaucipir signal across the entire cortex with the exception of postcentral, precentral, and paracentral gyri and anterior cingulate. **D** Surface map showing the percentages of subjects that are tau-positive for all FreeSurfer regions in B1. **E** Surface map showing the percentages of subjects that are tau-positive for all FreeSurfer regions in B2. **F** Surface map showing the percentages of subjects that are tau-positive for all FreeSurfer regions in B3

Similar to surface maps of studies 1 and 2, we observed that there were no regions of elevated flortaucipir uptake in B1 NFT score subjects (Fig. 6A). The elevated uptake was primarily restricted to the *Eτ* VOI, with the exception of bilateral parahippocampal gyrus for B2 NFT score subjects. There was an elevation in the middle temporal gyrus, sections of the medial orbitofrontal gyrus, and anterior cingulate for B2 NFT score subjects (Fig. 6B). We observed elevated uptake across the entire cortex sparing the precentral, postcentral, and paracentral gyri and the anterior cingulate for B3 NFT score subjects (Fig. 6C). Voxel-wise *z*-score surface maps yielded a similar pattern of elevated uptake (Fig. 6D–F).

## Discussion

In this study, we identified a population of patients with elevated flortaucipir signal in the early tau region (*Eτ* VOI) and low global AD signature-weighted neocortical VOI uptake. The subjects with elevated *Eτ* VOI signal and low AD signature-weighted neocortical VOI signal were 84% Aβ+, 59% *APOE ε4* carriers, and diagnosed as MCI or AD. (Fig. 2).

The quadrant-wise statistical analysis disclosed significantly different 18-month tau accumulation between *Eτ*−/AD_signature_− subjects compared to *Eτ+*/AD_signature_– for both AD_signature_ and *Eτ* VOIs (Fig. 3A, B). While there was a significant difference between subjects in *Eτ+*/AD_signature_– and *Eτ+*/AD_signature_+ for AD_signature_ SUVR, we did not observe a significant difference in 18-month *Eτ* VOI accumulation. *Eτ+*/AD_signature_– subjects had 18-month change in thickness values in-between *Eτ*−/AD_signature_− and *Eτ+*/AD_signature_+ (Fig. 3C). Furthermore, the 18-month change in MMSE (Fig. 3D), ADAS-Cog11, and FAQ values for *Eτ+*/AD_signature_– subjects were between *Eτ+*/AD_signature_+ and *Eτ*−/AD_signature_−, suggesting that *Eτ+*/AD_signature_– subjects may be intermediate stage subjects. These findings also suggest that *Eτ+*/AD_signature_– subjects might be at an earlier stage of AD pathology relative to *Eτ+*/AD_signature_+ subjects.

The performance of the *Eτ* VOI in identifying early-stage subjects was explored in the autopsy-confirmed cohort. We observed that 80% (12/15) of B2 pathology subjects, including 10/11 Braak IV cases, had an elevated *Eτ* SUVR in the absence of an elevated AD signature-weighted neocortical SUVR. In contrast, only 2/4 Braak III subjects had elevated *Eτ* VOI signal. While there was increased sensitivity in the detection of B2 pathology subjects using the *Eτ* VOI, 4 Braak VI subjects were misclassified as having no *Eτ* flortaucipir uptake. Similarly, 4 Braak V subjects were also misclassified. We suspect this might be due to the significant atrophy in these subjects, particularly within the early tau regions. It is widely reported that medial temporal regions are likely to be atrophied first in AD [40, 41]. Accordingly, the SUVR in these early tau regions might be lower compared to further downstream tau regions such as posterolateral temporal and parietal, which is where AD_signature_ mainly samples from. Although a small sample, this might suggest that the *Eτ* VOI, when compared to the AD_signature_ VOI, was sensitive at identifying Braak IV cases (Fig. 4B). While previous neuropathologic studies have demonstrated the correlation between neurofibrillary tangles and flortaucipir retention [42, 43], studies on flortaucipir PET images with autopsy confirmation in moderate sample sizes are limited. Accordingly, one strength of the current work is the identification of a positive signal with the *Eτ* VOI in a moderate sample of autopsy-confirmed intermediate Braak pathology subjects.

Our data suggests that PERSI might provide better sensitivity in identifying subjects that progress within 18 months. While the trends with cerebellum crustaneous were similar to the PERSI reference region, tau accumulation and cortical thinning were not significantly different between *Eτ–*/AD_signature_– and *Eτ+*/AD_signature_– groups. As previously published, the PERSI reference region had lesser variability in SUVR of controls compared to cerebellum crustaneous [33]. This increased variability necessitates a higher cutoff for *Eτ* VOI with cerebellum crustaneous reference region to provide the same confidence in the identification of elevated signal resulting in fewer subjects being identified as *Eτ+*/AD_signature_–.

*Eτ+*/AD_signature_– subjects were defined by elevation in *Eτ* VOI but not in AD_signature_. In order to determine whether there might be other regions also affected in *Eτ+*/AD_signature_– subjects, we sampled the entire cortex and created surface maps. Based on the surface maps (Fig. 5B, E), the involvement of medial structures such as the entorhinal cortex, fusiform, and parahippocampal gyri and a lateral structure (inferior temporal gyrus) was evident across the majority of *Eτ+*/AD_signature_– subjects. Widespread involvement of multiple regions was seen in *Eτ+*/AD_signature_+ subjects (Fig. 5C, F) with the absence of flortaucipir signal across the entire cortex for *Eτ–*/AD_signature_– subjects (Fig. 5A, D). The surface maps of the autopsy data showed similar findings, where the involvement of the entorhinal cortex, fusiform, parahippocampal, and inferior temporal gyrus was evident across the majority of B2 NFT score subjects. B2 NFT score subjects also showed elevated flortaucipir uptake in the sections of the medial orbitofrontal region and anterior cingulate gyrus (Fig. 6B, E). The pattern of elevation of flortaucipir retention was consistent with neuropathological Braak stage and with the proposed sensitivity of the *Eτ* region to B2 pathology.

This work has several limitations. Since not all participants with PET data have MRIs, particularly our autopsy study participants, we created a template space VOI. As the VOI is a template-based region, which is derived from anatomical MRI, it is relatively small and restricted to the cortex. Consequently, uptake might be reduced in cases of substantial temporal lobe atrophy or instances of misalignments to the MNI template. Subjects with high levels of tau and atrophy might lead to difficulties in quantification. The majority of the 8.7% (37/427) subjects excluded in this analysis have either high levels of tau or atrophy. Since we are primarily interested in subjects with early stages of tau accumulation, we believe the exclusion of these subjects does not affect our findings. Here, we used a data-driven AD_signature_ VOI to represent global tau uptake and a template-based *Eτ* VOI to characterize regional tau uptake. Not including a data-driven version of the early tau VOI might be another limitation of this work, however, some reports suggest similar spatial distribution patterns between data-driven and anatomical VOIs, particularly in early tau regions [29]. Furthermore, data-driven VOIs can rely heavily on the datasets used to develop them and may not be easily generalizable to other datasets.

Another limitation of the approach in this study is the inclusion of older Aβ− controls. While one might argue that the statistical difference observed is driven by the controls, it is important to note that the subjects were classified into quadrants agnostic to their clinical diagnosis or amyloid status. Furthermore, subject grouping based on the *Eτ* VOI may be sensitive to classifying controls as being tau negative.

The small number of Aβ+ cognitively normal subjects in our cohort is another limitation of our study. The prevalence of AD or MCI subjects in *Eτ+*/AD_signature_– may be slightly inflated due to the limited representation of Aβ+ controls. Although all Aβ+ controls were below the *Eτ* VOI cutoff in this data, it is routinely reported that cognitively normal Aβ+ subjects have elevated inferior temporal tau uptake [19, 35, 44]. One possibility for not observing this trend in this data might be the restrictive MMSE range (MMSE 29, 30) allowed in subjects classified as cognitively normal. We have imposed this strict MMSE threshold in order to obtain a clearer picture of tau levels in normal aging, considering the possibility that Aβ+ subjects with lower MMSE (e.g., 26 or 27) that might have been classified as cognitively unimpaired in other studies [19, 44] may actually be showing impairment consistent with the earliest stages of Alzheimer’s disease. This data suggests that *Eτ+*/AD_signature_– subjects present an intermediate level of progression between *Eτ*−/AD_signature_− and *Eτ+*/AD_signature_+ subjects. Accordingly, a natural extension of this work would be to explore if *Eτ+*/AD_signature_– subjects eventually convert to advanced disease subjects.

It is important to emphasize that the regions suggested in this work are not the only ones that might identify early AD subjects. Multiple studies have suggested individual temporal, composite, and temporo-parietal regions to detect similar subgroups of subjects [19, 20, 26–29, 35, 45, 46]. Jack et al. have shown that their early AD meta-ROI consisting of fusiform and posterior cingulate best separates cognitively normal Aβ− and Aβ+ subjects [27]. Likewise, Mishra et al. have found entorhinal cortex, lateral occipital cortex, inferior temporal, and amygdala as being important in differentiating between high and low tau preclinical AD subjects [47]. Johnson et al. report elevated tau uptake within the inferior temporal lobes of preclinical AD subjects, and Hanseeuuw et al. report a greater increase in tau levels within the inferior temporal cortex of individuals with increasing Aβ [25, 44]. We compared the *Eτ* VOI with FreeSurfer-derived regions of the inferior temporal lobe, composite of the fusiform and posterior cingulate (similar to early AD meta ROI) [27], and composite of the amygdala, entorhinal cortex, fusiform, parahippocampal, inferior temporal, and middle temporal gyri (similar to temporal meta-ROI) [26]. We observed similar differences in baseline SUVR, 18-month change in SUVR, cortical thickness, and MMSE. The performance of these regions varied slightly in detecting autopsy-confirmed Braak IV subjects. The inferior temporal lobe, *Eτ* VOI, and AD meta-ROI-like and early AD meta-ROI-like VOIs identified 11/11, 10/11, 9/11, and 7/11 Braak IV subjects, respectively (Fig. S4A-D). Since the early AD meta-ROI does not sample from the inferior temporal region, we think its performance in identifying B2 NFT score subjects is slightly lower. Lowe et al. have shown that using the AD-centric meta-ROI [26], they were able to identify autopsy-confirmed Braak IV and higher subjects [48].

In this work, we have shown that the *Eτ* VOI is not only sensitive in identifying Braak IV and higher subjects but also has good specificity (2/7 B1 NFT score subjects were identified). In addition, combining our *Eτ* and AD_signature_ VOI, we can differentiate Braak IV subjects. In separate cohorts, we have also shown that these subjects (identified by the E*τ* VOI) progress within 18 months in terms of tau accumulation, neurodegeneration, and cognition. Within the data presented here, on average, tau was predominantly restricted to the set of regions selected.

## Conclusion

The *Eτ* VOI identified subjects with elevated temporal but not global tau that were primarily Aβ+, *APOE ε4* carriers, and diagnosed as MCI or AD. *Eτ+*/AD_signature_– subjects had greater accumulation of tau, greater atrophy, and higher decline on MMSE, FAQ, and ADAS-Cog11 in 18 months compared to *Eτ*−/AD_signature_− subjects. Furthermore, the *Eτ+*/AD_signature_– subjects did not have elevated flortaucipir outside the medial/lateral temporal regions. Finally, the *Eτ* VOI accurately identified the majority of the intermediate NFT score subjects in our autopsy-confirmed data. As far as we know, this is the first study that presents a visualization of ante-mortem FTP retention patterns that at a group level agree with the neurofibrillary tangle staging scheme proposed by Braak. Based on these findings, we conclude that the *Eτ* VOI may be sensitive for detecting symptomatic subjects early in the course of Alzheimer’s disease.

## Supplementary Information

**Additional file 1: Supplementary Figure S1.**

**Additional file 2: Supplementary Figure S2.**

**Additional file 3: Supplementary Figure S3.**

**Additional file 4: Supplementary Figure S4.**

**Additional file 5: Supplementary Table S1.**

## Data Availability

Lilly provides access to all individual participant data collected during the trial, after anonymization, with the exception of pharmacokinetic or genetic data. Data are available to request 6 months after the indication studied has been approved in the USA and EU and after primary publication acceptance, whichever is later. No expiration date of data requests is currently set once data are made available. Access is provided after a proposal has been approved by an independent review committee identified for this purpose and after receipt of a signed data sharing agreement. Data and documents, including the study protocol, statistical analysis plan, clinical study report, and blank or annotated case report forms, will be provided in a secure data sharing environment. For details on submitting a request, see the instructions provided at www.vivli.org.
