# Supplementary figures and images for: Early tau detection in flortaucipir images: validation in autopsy-confirmed data and implications for disease progression
Source: Alzheimers Res Ther. 2023 Feb 28;15:41. doi: 10.1186/s13195-023-01160-6 (PMC9972744; doi:10.1186/s13195-023-01160-6)

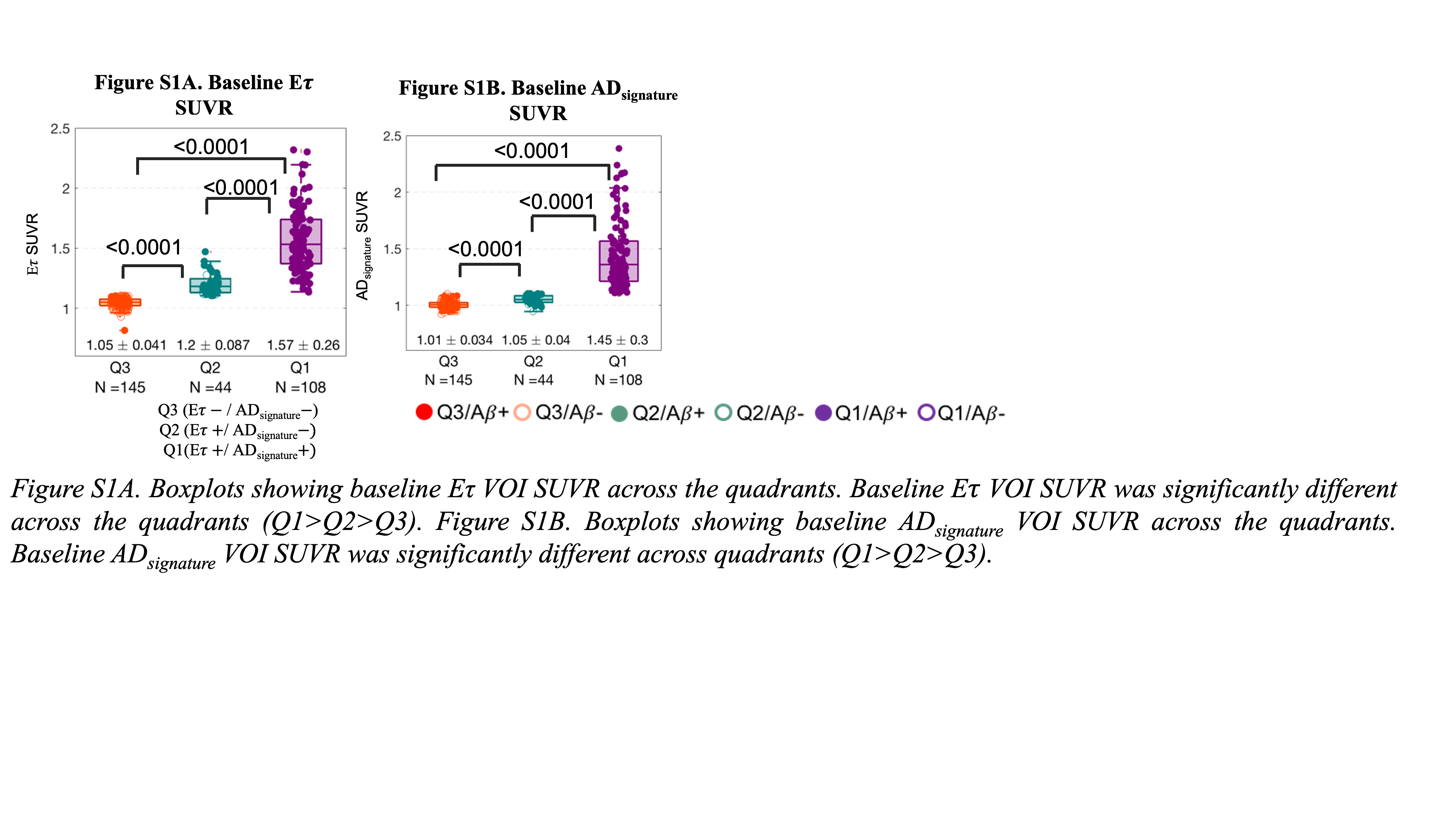

Supplement: Supplementary file 1 — Additional file 1: Supplementary Figure S1. [file 13195_2023_1160_MOESM1_ESM.tiff]

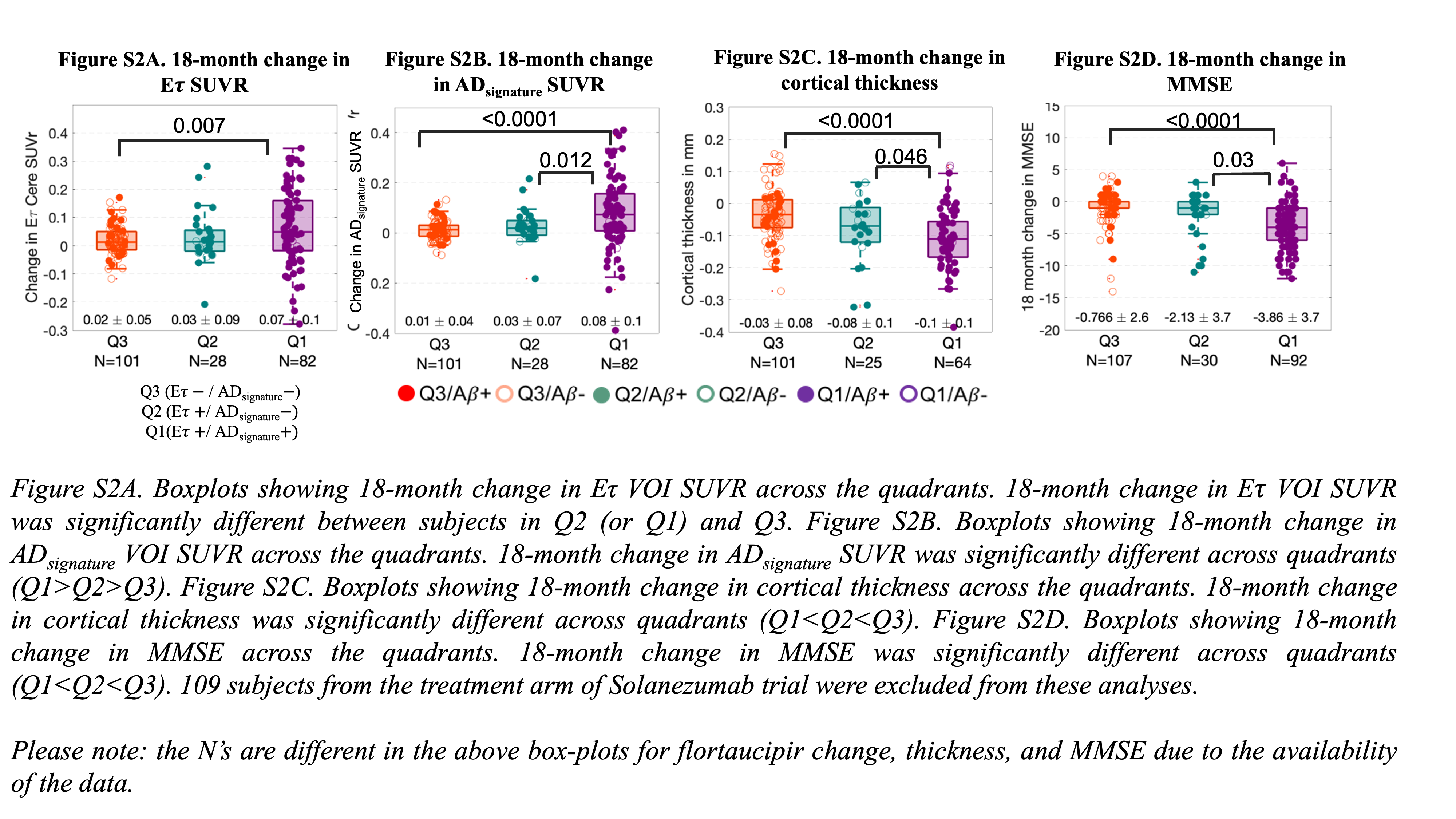

Supplement: Supplementary file 2 — Additional file 2: Supplementary Figure S2. [file 13195_2023_1160_MOESM2_ESM.tiff]

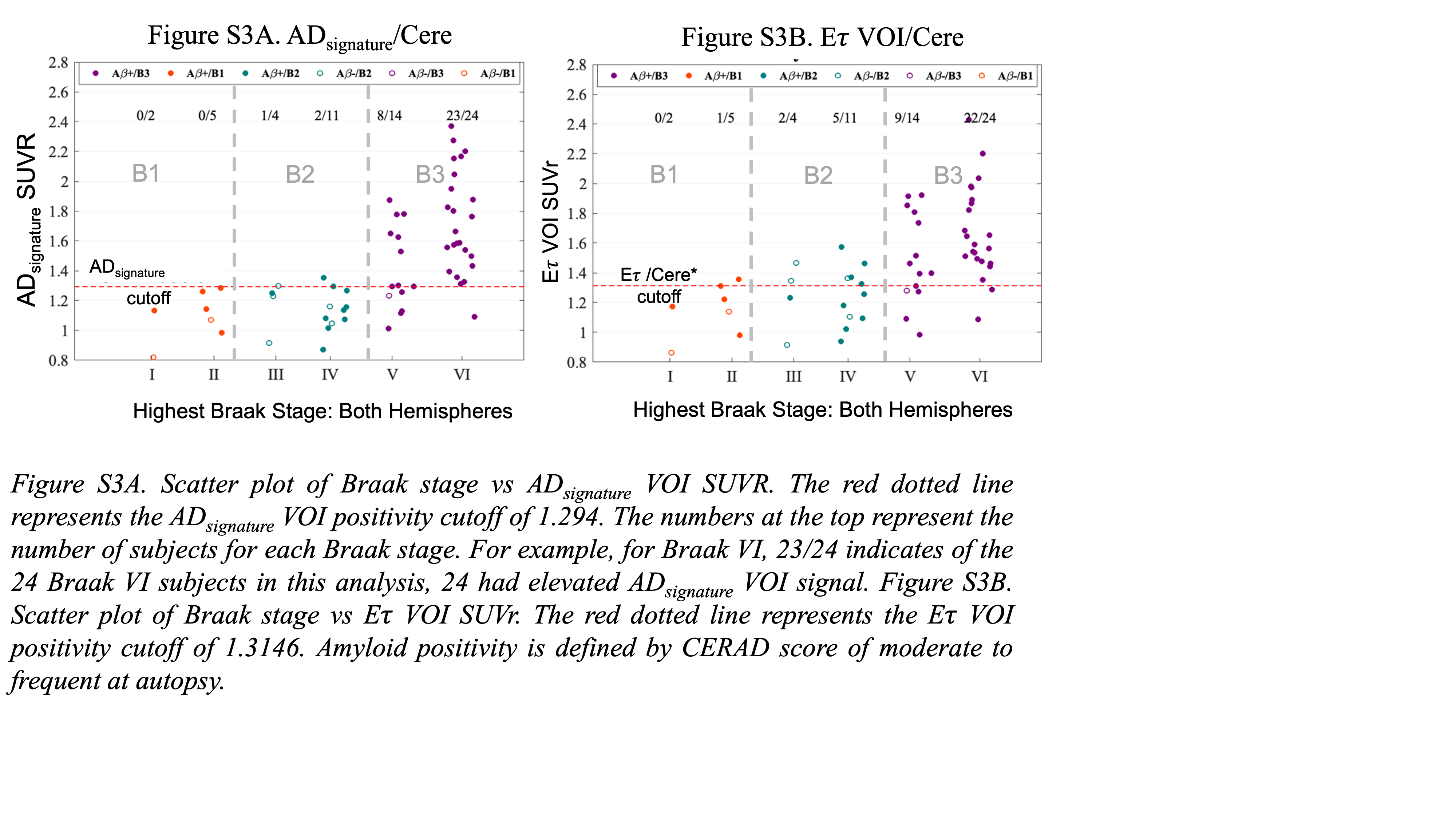

Supplement: Supplementary file 3 — Additional file 3: Supplementary Figure S3. [file 13195_2023_1160_MOESM3_ESM.tiff]

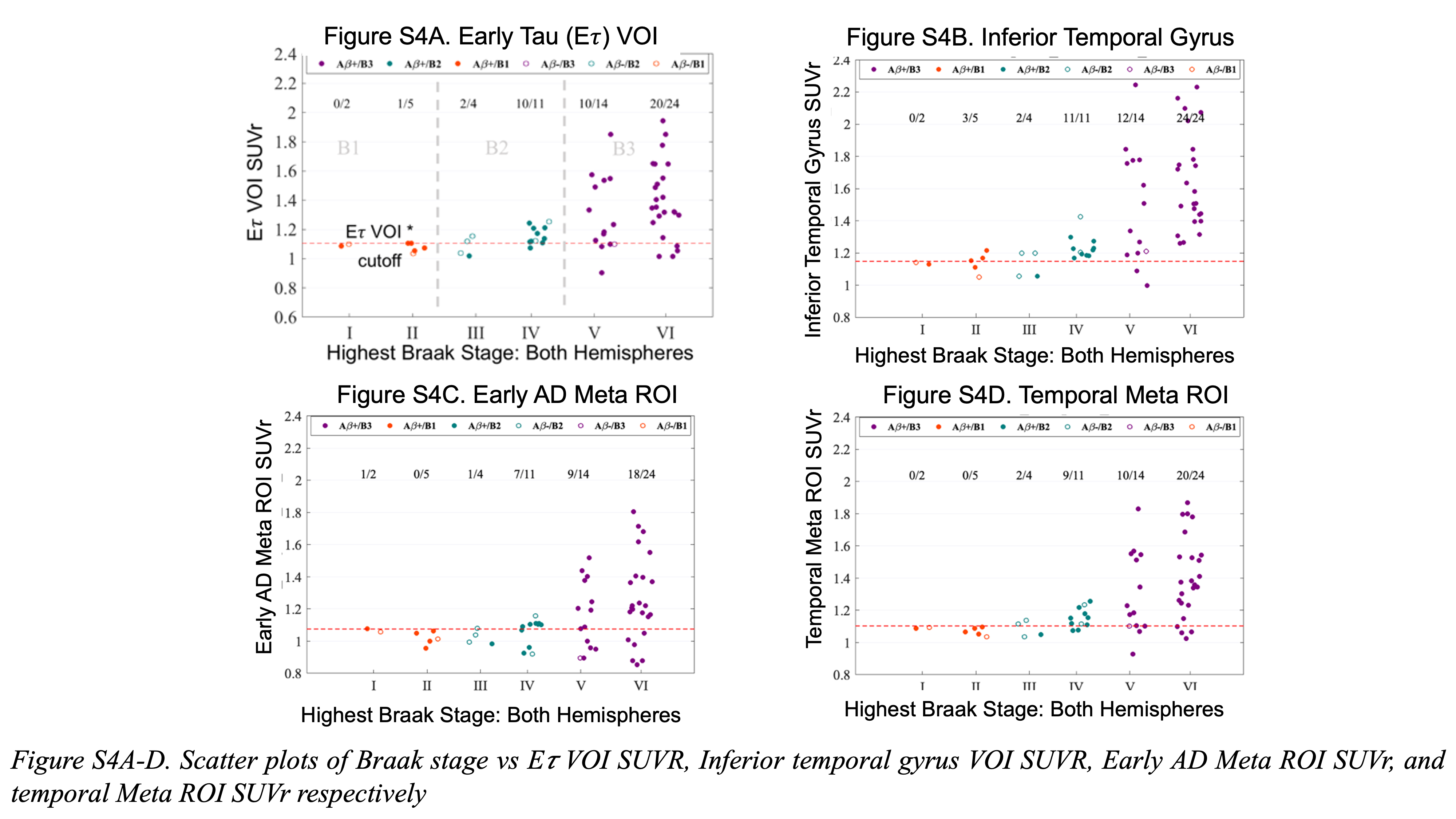

Supplement: Supplementary file 4 — Additional file 4: Supplementary Figure S4. [file 13195_2023_1160_MOESM4_ESM.tiff]

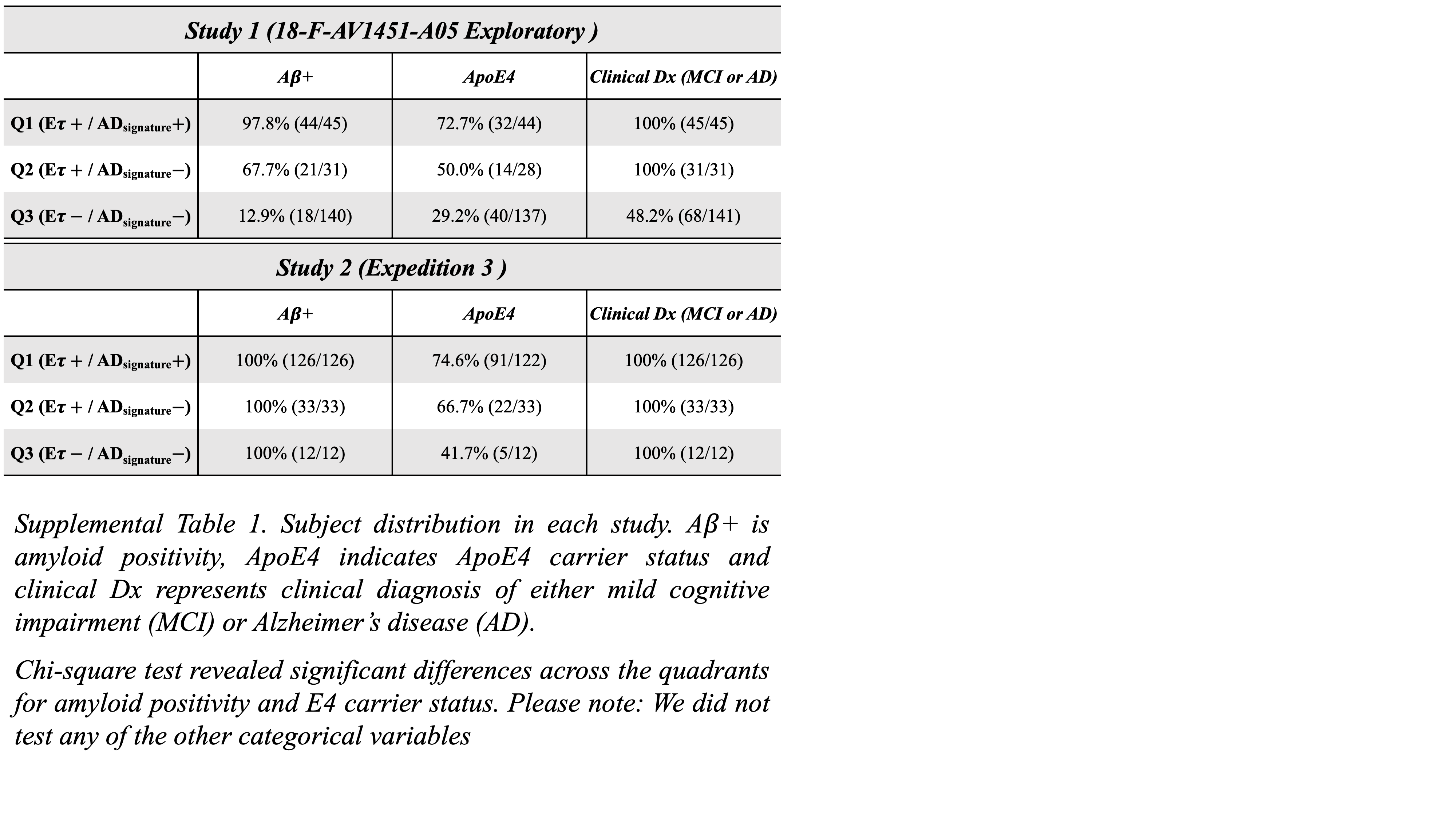

Supplement: Supplementary file 5 — Additional file 5: Supplementary Table S1. [file 13195_2023_1160_MOESM5_ESM.tiff]
